# Whole genome analysis of the koa wilt pathogen (*Fusarium oxysporum* f. sp. *koae*) and the development of molecular tools for early detection and monitoring

**DOI:** 10.1186/s12864-020-07156-y

**Published:** 2020-11-04

**Authors:** John T. Dobbs, Mee-Sook Kim, Nicklos S. Dudley, Ned B. Klopfenstein, Aileen Yeh, Robert D. Hauff, Tyler C. Jones, R. Kasten Dumroese, Philip G. Cannon, Jane E. Stewart

**Affiliations:** 1 Colorado State University, Department of Agricultural Biology, 1177 Campus Delivery, Fort Collins, CO 80523 USA; 2 USDA Forest Service, Pacific Northwest Research Station, 3200 SW Jefferson Way, Corvallis, OR 97331 USA; 3 Hawai‘i Agriculture Research Center, Maunawili Research Station, Oahu, HI USA; 4 USDA Forest Service, Rocky Mountain Research Station, 1221 South Main Street, Moscow, ID 83843 USA; 5 Division of Forestry and Wildlife, Department of Land and Natural Resources, 1151 Punchbowl Street, Room 325, Honolulu, HI 96813 USA; 6 USDA Forest Service, Forest Health Protection, 1323 Club Drive, Vallejo, CA 94592 USA

**Keywords:** *Fusarium oxysporum*, Lineage-specific DNA, Virulence genes, PCR primer, Haplotypes, *Acacia koa*

## Abstract

**Background:**

Development and application of DNA-based methods to distinguish highly virulent isolates of *Fusarium oxysporum* f. sp. *koae* [*Fo koae;* cause of koa wilt disease on *Acacia koa* (koa)] will help disease management through early detection, enhanced monitoring, and improved disease resistance-breeding programs.

**Results:**

This study presents whole genome analyses of one highly virulent *Fo koae* isolate and one non-pathogenic *F. oxysporum* (*Fo*) isolate. These analyses allowed for the identification of putative lineage-specific DNA and predicted genes necessary for disease development on koa. Using putative chromosomes and predicted gene comparisons, *Fo koae*-exclusive, virulence genes were identified. The putative lineage-specific DNA included identified genes encoding products secreted in xylem (e. g., *SIX1* and *SIX6*) that may be necessary for disease development on koa. Unique genes from *Fo koae* were used to develop pathogen-specific PCR primers. These diagnostic primers allowed target amplification in the characterized highly virulent *Fo koae* isolates but did not allow product amplification in low-virulence or non-pathogenic isolates of *Fo*. Thus, primers developed in this study will be useful for early detection and monitoring of highly virulent strains of *Fo koae*. Isolate verification is also important for disease resistance-breeding programs that require a diverse set of highly virulent *Fo koae* isolates for their disease-screening assays to develop disease-resistant koa.

**Conclusions:**

These results provide the framework for understanding the pathogen genes necessary for koa wilt disease and the genetic variation of *Fo koae* populations across the Hawaiian Islands.

**Supplementary information:**

**Supplementary information** accompanies this paper at 10.1186/s12864-020-07156-y.

## Background

Intraspecific genomic comparisons between pathogenic and non-pathogenic fungal isolates can elucidate key factors required for pathogenicity in plant hosts [1, 2]. These factors include unique genes that are not only vital for the pathogen to cause disease, but can also provide tools to differentiate pathogens and non-pathogens for detection and monitoring. Variation in genome size, predicted effectors, and unique genome regions have been used previously to differentiate plant pathogens from saprophytic non-pathogens [3–5]. Just as the hosts exhibit variation in resistance, fungal pathogens (e.g., *Fusarium oxysporum*) also exhibit variation in virulence [6]. Previous analyses of *F. oxysporum* f. sp. *lycopersici* and *F. oxysporum* f. sp. *radicis*-*cucumerinum* chromosomes identified conditionally dispensable chromosomes containing virulence factors that could be transferred between compatible strains through heterokaryon formation [4, 7]. Other previous studies found that these virulence factors were encoded by diverse genes. *Fusarium* transcription factors and their corresponding virulence genes, such as those encoding proteins secreted in xylem (*SIX*), were found to be essential for pathogenesis and high virulence in most *F. oxysporum* formae species [8–11]. Based on carbohydrate-active enzyme analysis, the copy number and types of plant cell wall-degrading enzymes have been found to be important for pathogenesis of *F. oxysporum* formae species in legumes [12]. In addition, phytotoxic secondary metabolites and associated transporters (i.e., fusaric acid, fumonisin B1, and beauvericin) have been identified as important for pathogenesis in the *F*. *oxysporum* f. sp. *cubense* [12, 13], and transposable elements (i.e., miniature impala elements) have been documented to enhance effector diversity [14–16]. These factors help distinguish pathogenicity and/or virulence in *Fusaria* and could also provide a basis for early detection of pathogens which would facilitate disease management programs through efforts to reduce pathogen spread to uninfested sites.

*Fusarium oxysporum* is a cosmopolitan, ascomycete fungus with observed functions that range from an innocuous soil saprophyte to a catastrophic plant pathogen in important agricultural crops and ecologically important tree species [17]. In addition to these diverse functions, *F. oxysporum* is also known to display high levels of host-specificity. These highly host-specific pathogenic strains are classified into formae speciales (forma specialis singular) that exhibit high genomic plasticity, which has been attributed to effector profiles housed on one to many conditionally dispensable, lineage-specific accessory chromosomes, where the vast majority of genomic variation exists, in comparison to a set of shared “core” chromosomes containing essential genes for normal cellular function [16, 18–20]. These lineage-specific chromosome(s) house genes involved in virulence and/or evasion of plant host detection and have been described as repeat- and transposon-rich [1, 3]. Comparisons of these virulence genes through whole-genome analyses are useful for distinguishing pathogens from non-pathogens on the susceptible host of a specific *F. oxysporum* forma specialis [1]. In this study, we analyzed and compared the genomes of pathogenic and non-pathogenic *F. oxysporum* strains, which were both isolated from the roots of *Acacia koa* A. Gray (koa).

Gardner (1980) described Fusarium wilt disease in low- to mid-elevation, below 610 m (2000 ft), forests in the Hawaiian archipelago affecting koa, an endemic tree species [21, 22]. Koa trees are ecologically, economically, and culturally valuable to Hawai‘i. Koa is the second most abundant tree in Hawai‘i’s forests and many of Hawai‘i’s indigenous flora and fauna rely on koa for food and shelter [21]. Due to koa’s importance to Hawai‘i, efforts have been increased to reincorporate koa into lowland forests, where its populations have declined [23]. Currently, koa wilt disease [caused by *F. oxysporum* f. sp. *koae* (*Fo koae*)] represents the predominant threat to koa across its native range [24].

Management methods for koa wilt disease include fumigation and pesticides that are environmentally harmful and economically costly when used in forests and/or timber stands. As a result, current efforts are focused primarily on producing disease-resistant seedlings derived from seed collected from natural stands [23]. To find naturally occurring resistance to the pathogenic strains of the fungus (*Fo koae*), wild-collected seed is used for extensive disease resistance-screening assays in the greenhouse [25]. These screening trials are costly and require diverse pathogenic *Fo koae* strains to ensure that the disease resistance is robust in the selected germplasm. Consequently, these disease resistance-screening programs would greatly benefit from faster, more cost-effective strategies for screening pathogenic *Fo koae* isolates. This screening process is further complicated by considerations that pathogenic fungi can evolve quickly; much faster than their plant hosts [1, 26, 27]. For this reason, new, genetically diverse strains of the pathogen must be continually incorporated in screening programs to help ensure that disease resistance remains stable against newly evolved or introduced strains of the pathogen in Hawai‘i’s forest and agricultural lands. Collecting, isolating, and characterizing pathogenic *Fo koae* isolates is an arduous and time-consuming process that requires 3-month greenhouse virulence assays to confirm pathogenicity. Pathogen-specific, PCR primers would allow for rapid detection of pathogenic *Fo koae* strains, which would expedite collections of new pathogenic strains for disease resistance-breeding programs to meet the needs of growers and land managers for restoration and/or timber purposes.

Objectives for this study were to 1) conduct and analyze whole genome sequences of one highly virulent *Fo koae* isolate and one non-pathogenic *F. oxysporum* (*Fo*) isolate both collected from koa; 2) identify virulence-associated genes with putative roles in koa wilt disease; 3) use the identified differences to develop *Fo koae*-specific PCR primers; and 4) test the robustness of the developed primers to identify highly virulent *Fo koae* isolates among uncharacterized *F. oxysporum* populations.

## Results

### Whole genome sequencing and assembly

To conduct whole genome comparisons, one pathogenic *Fo koae* isolate (44) and one non-pathogenic *Fo* isolate (170) were whole genome sequenced using Illumina TruSeq short-read sequencing (150 bp). Libraries resulted in 397,390,528 reads with 1245x coverage for pathogenic isolate *Fo koae* 44 and 385,805,256 reads with 1150x coverage for non-pathogenic isolate *Fo* 170. De novo assembly of both *Fo koae* 44 and *Fo* 170 identified genome sizes of 48 and 50 Mb, respectfully. The QUAST report metrics are shown in Table 1.

**Table 1:** QUAST assembly statistics of the SPAdes de novo genome assembly of the pathogenic *Fusarium oxysporum* f. sp. *koae* 44 (*Fo koae* 44) and non-pathogenic *F. oxysporum* 170 (*Fo* 170) isolates

	***Fo koae*** 44	***Fo*** 170
Genome size (Mb)	48,183,513	50,628,816
Contig Number	492	964
Scaffold Number	330	910
N50	721,547	562,269
GC%	47.5	47.2

### Whole genome phylogenetic analysis

A whole-genome maximum likelihood phylogeny was constructed to elucidate the evolutionary relationships of *Fo koae* 44 and *Fo* 170 compared with other *Fusarium* spp. and *F. oxysporum* formae speciales (Additional File 9). The phylogeny showed that both *Fo koae* 44 and *Fo* 170 grouped into a well-supported [Bootstrap (BS) = 100] clade with other *F. oxysporum* formae speciales (Additional File 1). Interestingly, both isolates grouped into separate sub-clades with distinct *F. oxysporum* formae speciales, rather than grouping together (Additional File 1). *Fo koae* 44 clustered in a sub-clade (BS =100) that included *F. oxysporum* f. sp. *cubense* race 1 and *Fo* 170 clustered in a sub-clade (BS =100) with *F. oxysporum* f. sp. *gladioli*. This result suggests that these isolates were indeed *F. oxysporum* and that *Fo koae* 44 and *Fo* 170 were sufficiently, genetically different to allow comparison for identifying lineage-specific DNA necessary for pathogenicity in koa*.*

### Genomic analyses of *Fo koae* 44 and *Fo* 170

**Fig. 1:**
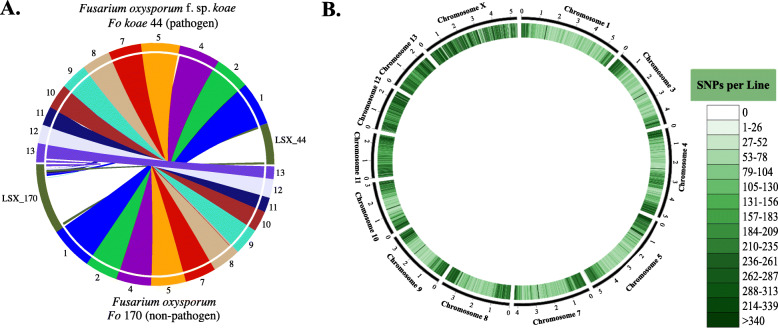
**a** Synteny map showing synteny between each of 11 putative core chromosomes that were mapped to *Fusarium oxysporum* f. sp. *lycopersici* (reference strain). Most of the putative twelfth chromosome (putative lineage-specific DNA) denoted as LSX_44 for *F. oxysporum* f. sp. *koae* 44 (*Fo koae* 44) and LSX_170 for *F. oxysporum* 170 (*Fo *170) does not show synteny between the pathogen and non-pathogen and did not map to the reference strain. This lack of synteny on the putative twelfth chromosome is indicative of lineage-specific DNA, putatively containing conditionally dispensable, accessory chromosomes. **b** Distribution of variants (single nucleotide polymorphisms, SNPs) comparing the putative chromosomes of the koa wilt pathogen *Fo koae* 44 and non-pathogenic *Fo* 170. Darker bars indicate more SNPs identified in that region. Putative chromosomes 11, 12, 13, and putative lineage-specific DNA (ChromosomeX; LSX) show the highest SNP densities

**Fig. 2:**
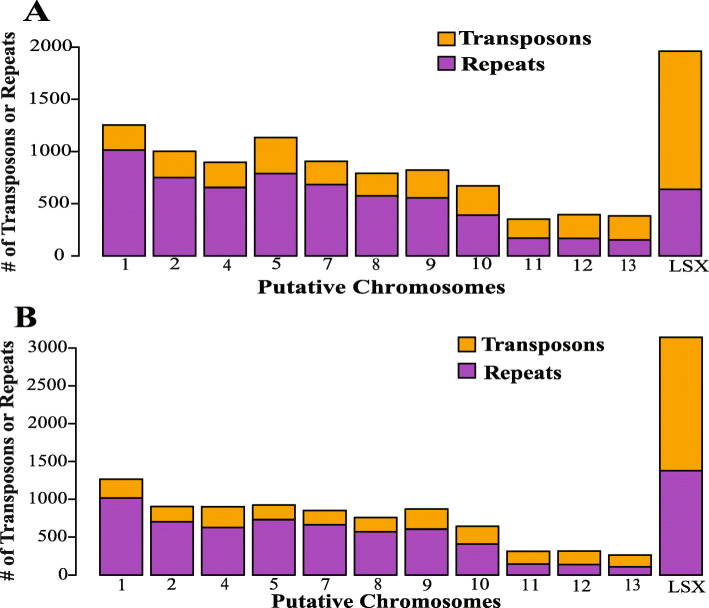
**a** Distribution of transposons (orange bars) and repeats (purple bars) across the putative chromosomes and putative lineage-specific DNA (LSX) of *Fusarium oxysporum* f. sp. *koae* (*Fo koae* 44; koa wilt pathogen). **b** Distribution of transposons (orange bars) and repeats (purple bars) across the putative chromosomes LSX in *Fusarium oxysporum* (*Fo* 170). Majority of the transposons were identified in the LSX

Genomic sequences of *Fo koae* 44 and *Fo* 170 were aligned with reference genome sequences of a well-characterized *F. oxysporum* f. sp. *lycopersici* isolate (GenBank accession AAXH00000000.1) to identify regions of highest dissimilarity and identify lineage-specific DNA, with potential use for pathogen-specific primer development. Based on the reference genome*,* 11 putative “core” chromosomes shared synteny with *Fo koae* 44 and *Fo* 170. Putative lineage-specific DNA (LSX) was identified in each genome that did not map to the genome of *F. oxysporum* f. sp. *lycopersici* or show synteny with each other (*Fo koae* 44 and *Fo *170) or genomes of other *F. oxysporum* formae speciales (Fig. 1a). Low-coverage sequences and non-*Fusarium* DNA were discarded to remove possible DNA contamination. The average nucleotide identity (ANIm) between *Fo koae* 44 and *Fo* 170 was 96.5%. The remaining 3.5% genetic dissimilarity is largely due to the identified LSX. Further analyses comparing the putative chromosomes of *Fo koae* 44 and *Fo* 170 indicated that variants (SNPs and indels) were localized on specific chromosomes (LSX_44 and LSX_170) and at chromosome ends (Fig. 1b). Putative core chromosomes 1, 2, 4, 5, 7, 8, 9, and 10 were the most similar with identified variants localized on chromosome ends, likely due to higher recombination observed at telomeres or assembly errors [20, 28]. Putative chromosomes 11, 12, 13, and LSX had the most variation (Fig. 1b). Analyses of the distribution of repeats and transposons determined that *F. oxysporum* transposable elements (e.g., *Foxy*, *Skippy*, *Impala*, *Hop*, *Mariner*, *Hat*, and *Helitron*) occurred on the LSX of both *Fo koae* 44 and *Fo* 170 [29]. The average core chromosome was comprised of 3 and 4% transposable elements and repeats in *Fo koae* 44 and *Fo* 170, respectively. In contrast, the transposable elements and repeats comprised 19 and 20% of LSX for *Fo koae* 44 and *Fo* 170, respectively (Fig. 2a & b). This localization of transposable elements provides evidence that differences within the LSX are likely attributable to one or more conditionally dispensable chromosome(s) in both *Fo koae* 44 and *Fo* 170.

### Genome annotation

Using the Maker pipeline, 15,380 and 15,763 transcripts and corresponding proteins were predicted for *Fo koae* 44 and *Fo* 170, respectively, for use in identifying genes associated with pathogenicity in koa wilt disease. Using OrthoVenn2, 1760 non-orthologous proteins from 78 gene clusters and 1576 single genes, were identified as exclusive to *Fo koae* 44 when compared to *Fo* 170. In comparison, *Fo* 170 had 2123 exclusive non-orthologous proteins from 137 gene clusters and 1780 single genes. *Fo koae* 44 had enrichment of gene ontology (GO) terms for nucleic acid binding (Additional File 2), whereas *Fo* 170 had enrichment of GO terms for GTP binding, regulation of transcription from RNA polymerase II promoter during meiosis, sporulation resulting in formation of a cellular spore, and regulation of DNA-template transcription (Additional File 3). The 13,500 protein clusters that were shared between *Fo koae* 44 and *Fo* 170 had no identified GO enrichment.

Using InterProScan, 16,429 and 16,487 putative protein family domains were identified in *Fo koae* 44 and *Fo* 170, respectively. Of these putative proteins, 30 were unique to *Fo koae* 44, and 32 were unique to *Fo* 170. Interestingly, a sterigmatocystin biosynthesis gene was identified as unique in *Fo koae* 44. Of further note is that the genome of *Fo* 170 also encoded some proteins involved in pathogenesis, including oxidoreductases (i.e., monooxygenase) and putrescine biosynthesis.

Using the antiSMASH database, the genes associated with secondary metabolites were similar in both genomes of *Fo koae* 44 and *Fo* 170; however, variation did exist in gene copy number (Additional File 4). Non-ribosomal peptide synthetases (NRPS), NRPS-like, and indoles had higher gene copy numbers for *Fo koae* 44. *Fo* 170 had two more copies of genes associated with the production of the *Fusarium* mycotoxin enniatin.

Similar results were observed from genes identified using the dbCAN2 database, except that two copies of a glycoside hydrolase, GH13_22 (characterized glucan synthase), were found in *Fo koae* 44, but not in *Fo* 170 [30]. In *Fo* 170, two gene copies encoding proteins GH141 (α-L-fucosidase or xylanase), one copy encoding GH30 (endo-β-1,4-xylanase or β-glucosidase), and one copy encoding GH43_12 (a glycoside hydrolase involved in hydrolysis and/or rearrangement of glycosidic bonds; arabinosidase) were identified.

Using the PHI-base, shared virulence-associated genes that were identified in both *Fo koae* 44 and *Fo* 170 include the following: transport associated genes, such as the ATP-binding cassette (*Gpabc1*; PHI:258); effectors, such as the *Ace1* (PHI:325) and *Mocdip1* (PHI:3213); *Fusarium*-specific transcription factors, such as the *Ftf2* (PHI:5484) and the *SIX* gene (*SGE1*; PHI:3168); toxin detoxifying genes, such as the *GzmetE* (PHI:355) and *Tom1* (PHI:438); a chitin synthase gene, *CHS2* (PHI:336); and a beta-1,3-glucanosyltransferase, *GAS1* (PHI:522). Nine virulence-associated genes were identified as unique to *Fo koae* 44. These genes encoded multiple *Fusarium* transcription factors, mitogen-activated protein kinases, and ATP-binding cassette (ABC) transporters. The *SIX1* and *SIX6* genes, were identified on the LSX of *Fo koae* 44, but were absent in the *Fo* 170 genome.

These *Fo koae* 44 virulence-associated genes showed sequence similarity to other *F. oxysporum* formae speciales (GenBank Accession numbers in Additional Files 10 and 5B). The *SIX1* gene of *Fo koae* 44 showed highest sequence similarity with *F. oxysporum* f. sp. *fragariae* (85%) and the *SIX6* gene showed highest sequence similarity to *F. oxysporum* f. sp. *pisi* (97%) and *F. oxysporum* f. sp. *cucumerinum* (97%). There were seven virulence-associated genes identified as unique to *Fo* 170 that consisted of signaling proteins, ATP binding cassette transporter, and one of unknown function but related to developing root rot symptoms (*PEP2*).

### *Fo koae*-specific PCR primer development

To increase the likelihood of developing a pathogen-specific primer, two approaches were used to classify genomic regions and genes unique to *Fo koae* 44 for PCR primer development. First, unique genomic regions, that may be non-coding DNA sequences, were identified in *Fo koae* 44. Second, predicted transcripts unique to *Fo koae* 44 were identified.

To identify unique genomic regions, *Fo koae* 44 chromosomes were aligned to chromosomes of *Fo* 170, other *Fusarium* spp., and *F. oxysporum* formae speciales obtained from GenBank (Additional File 9). Analysis of these alignments identified a total of 445 DNA sequences unique to *Fo koae* 44. Of these 445 DNA sequences, 33 had no BLAST hits to the NCBI database, and these 33 DNA sequences were selected for design of PCR primers.

To identify unique genes, *Fo koae* 44 and *Fo* 170 were analyzed using OrthoVenn2. One thousand seven hundred sixty-two predicted proteins were identified as exclusive to *Fo koae* 44. The corresponding transcripts were BLASTed to the NCBI database and 137 of these transcripts had no BLAST hits. These 137 transcripts were also selected for design of PCR primers.

Taken together, each of the 33 unique genomic DNA sequences and the 137 predicted transcripts (ca. 170 genome regions) were BLASTed against the chromosomes to determine which chromosome contained the selected sequence target for potential amplification. From these sequences, 3750 primers were developed in silico. Of those, 35 primer pairs had no non-target hits in silico through NCBI primer BLAST. These primers were tested on characterized pathogenic *Fo koae* isolates and non-pathogenic *F. oxysporum* (Table 2), *F. commune* (FO21 and 85), and *F. proliferatum* (1 and 80) isolates; all but one *F. commune* isolate (FO21) were collected from koa. Of the 35 primer pairs, 7 (one designed to amplify the “core” chromosome 2 and 6 designed to amplify the LSX) did not amplify DNA from non-target species. The 6 primers designed to amplify the LSX did not result in amplification using DNA from *Fo* 170 or other characterized non-pathogenic *F. oxysporum*, *F. commune*, and *F. proliferatum* isolates. The primer developed on the “core” chromosome 2 (P4) resulted in amplification of DNA from two of the characterized non-pathogenic isolates (72 and 81). Of the primer pairs that did not amplify non-target *Fusarium* species, 2 of primer pairs P4 (“core”) and P6 (LSX) were individually subjected to BLAST against the NCBI database to further test primers in silico for specificity (Additional File 5A). The “core” chromosome primer pair P4 had BLAST hits to other *Fusarium* spp. P4 forward and reverse primers had hits of 20 out 20 nucleotides to three genomes of *F. xyrophilum*, and 15 out of 20 nucleotides to *F. fujikuroi* and *F. graminearum* genomes, indicating this genomic region is likely found in other *Fusarium* spp. The LSX primer pair P6 also had partial BLAST hits to other *Fusarium* spp. in GenBank, including *F. venenatum*, *F. fujikuroi*, and *F. graminearum*, and the forward and reverse primers had 19 and 11 out of 20 nucleotides hits, respectively, to *F. culmorum* (Additional File 5B). Therefore, further testing of these primers, P4 and P6, on a broader suite of *Fusarium* spp. is warranted.

**Table 2:** *Fusarium oxysporum* f. sp. *koae* and *F*. *oxyporum* isolates that have been screened for pathogenicity through greenhouse virulence assays at the Hawai’i Agriculture Research Center

Isolate ID	Virulence	% Mortality*	Collection Site (Island)
44	High	95	Hawai’i
78	High	92	Kauai
77	High	80	Kauai
79	High	72	Kauai
0540 K	Moderate	70	Hawai’i
90	Moderate	68	Oahu
20	Moderate	66	Hawai’i
17	Moderate	62	Hawai’i
166	Moderate	60	Hawai’i
76	Moderate	60	Kauai
8	Moderate	58	Hawai’i
34	Low	12	Hawai’i
72	Nonpathogenic	8	Kauai
27	Nonpathogenic	8	Maui
53	Nonpathogenic	4	Maui
81	Nonpathogenic	4	Kauai
170	Nonpathogenic	0	Maui

*% mortality of 24 inoculated seedlings per isolate displaying foliar wilt, chlorosis, and/or necrotic symptoms (Dudley et al. 2007; Dudley et al. 2017)

### Field isolate collection

To test the specificity of primers P4 and P6 on a novel population of *Fusarium* isolates, root samples were collected from symptomatic koa trees. Root samples from 13 of the 14 sites surveyed in Hawaiʻi yielded *Fusarium* isolates. A total of 359 *Fusarium* isolates, including *F. oxysporum*, *F. solani*, *F. proliferatum*, *F. concolor*, *F. lateritium*, *F. commune*, and *F. fujikuroi* (based on *tef1* sequence data; GenBank Accession numbers and locations in Additional File 11), were collected from 100 symptomatic trees (70.4% of surveyed symptomatic trees) across the three islands (Additional File 6). Most of the *Fusarium* isolates were obtained from Maunawili, Kalopa, Wood Valley, and Pa’auilo, respectively. The least represented sites were Kona, Wung Ranch, and Kaiwiki, respectively.

### Genetic characterization of field-collected isolates

**Fig. 3:**
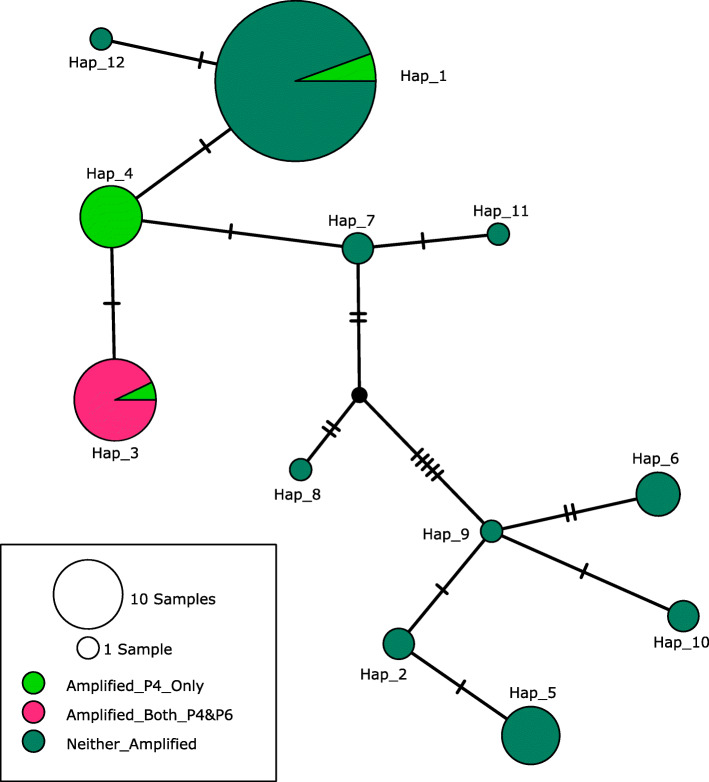
RNA polymerase II second largest subunit (*rpb2*) haplotype network of 12 haplotypes identified from 100 *Fusarium oxysporum* and *F. oxysporum* f. sp. *koae* isolates including 84 untested, field-collected isolates and 16 characterized isolates of high, moderate, and low virulence and non-pathogenic. All highly virulent isolates clustered in the Hap_3 haplotype. Moderate and low virulence isolates clustered in the Hap_1 haplotype. The characterized non-pathogenic isolates clustered in the Hap_2, Hap_4, and Hap_6 haplotypes. The r*pb2* haplotype network is overlaid with amplification from “core” chromosome primer pair P4 (designated by light green) and LSX primer pair P6 (designated in pink). Only the Hap_3 haplotype yielded amplification product with the LSX primer pair P6 from DNA of all highly virulent *F. oxysporum* f. sp. *koae* (*Fo koae*) and six uncharacterized isolates. Every isolate that amplified with the LSX primer pair also amplified with the “core” primer pair. “Core” chromosome primers (P4) produced amplification from three haplotypes including all highly virulent isolates in Hap_3 and two non-pathogenic isolates in Hap_4

To genetically characterize field-collected isolates with known virulence isolates, the *rpb2* was sequenced for isolates from individual trees. Sequencing of *rpb2* was conducted on 100 isolates, including 16 characterized for pathogenicity (4 highly virulent, 6 moderately virulent, 1 low-virulence, and 5 non-pathogenic isolates) and 84 field-collected isolates (Additional File 7). Variation at the *rpb2* locus was used to construct a TCS haplotype network that resulted in 12 haplotypes (Fig. 3).

The haplotype network of the *rpb2* revealed that the characterized highly virulent isolates (**44**, 77, 78, 79, 90, and 166) belonged to the same haplotype, Hap_3 (Fig. 3). Moderately virulent isolates clustered within haplotypes Hap_1 and Hap_3, while the low-virulence isolate clustered in Hap_1. Non-pathogenic isolates clustered within four haplotypes, Hap_1, Hap_2, Hap_4, and Hap_6. The developed primer pair P4, developed to amplify DNA from “core” chromosome 2, amplified DNA from 3 of the 12 *rpb2* haplotypes (Hap_1, Hap_3, and Hap_4), whereas the developed primer pair P6, developed to amplify LSX DNA, only amplified DNA from one haplotype (Hap_3) (Fig. 3). P4 produced amplification of DNA from highly virulent and moderately virulent isolates; however, P4 also amplified DNA from two non-pathogenic isolates (72 and 81) in Hap_4. DNA from uncharacterized isolates in Hap_1, Hap_3, and Hap_4 was amplified using the P4 primer pairs. The P6 primer pair did not produce amplification using DNA from non-pathogenic or lowly virulent isolates. The P6 primer pair amplified DNA from all but one uncharacterized isolate in Hap_3.

## Discussion

Plant pathogenic fusaria have been assessed for interspecific genetic variation to identify genomic features important for plant host infection [1, 2]. In addition, intraspecific genetic variation is a powerful tool to identify potential mechanisms for disease development by pathogenic fungi [31, 32]. This study presents a whole genome comparison between two *F. oxysporum* isolates collected from koa roots, one pathogenic (*Fo koae* 44) and one non-pathogenic (*Fo* 170) isolates. Through whole genome sequencing, genomic features were assessed bioinformatically for features that distinguish the pathogen from the non-pathogen. Similar to other intraspecific comparison studies, the two genomes in this study were very similar [3]. They shared 96% average nucleotide identity (ANIm), indicating that they belong to the same species [33]. The constructed whole-genome phylogeny also confirmed that these isolates were among the *F. oxysporum* species complex (Additional File 1). Synteny analysis identified that the putative chromosomes in both isolates were syntenic amongst the 11 shared chromosomes of the well-characterized *F. oxysporum* f. sp. *lycopersici* isolate [18]. Analysis of the syntenic chromosomes coincided with a recent study that observed higher recombination at the telomeres and amongst smaller chromosomes, while larger chromosomes were more conserved [28]. A non-syntenic “chromosome” was identified in both genomes, which is likely where most of the genes necessary to differentiate the koa pathogen are localized (Fig. 1b). Genome size has been shown to be a putative indicator of pathogenicity in filamentous fungi. Compared to their non-pathogenic counterparts, pathogens generally utilize larger genomes, which is associated with the presence of conditionally dispensable, lineage-specific accessory chromosomes [19]. These accessory chromosomes are known to be ca. 2 Mb, and they frequently house effectors necessary for disease development or evasion of host detection [4]. Despite these reports, this study found that the non-pathogen (*Fo* 170) had a slightly larger genome compared to the pathogen (*Fo koae* 44) at 50 and 48 Mb, respectively. Though *Fo* 170 had a larger genome, this study identified unique genes and DNA sequences, most notably the well-characterized secreted in xylem effectors (*SIX* genes), that were exclusive to *Fo koae* 44. Most of these unique genomic features of *Fo koae* 44 were identified amongst the LSX.

As *F. oxysporum* does not have an observed teleomorphic life stage, it relies on asexual means of recombination. As such, conditionally dispensable, accessory chromosomes, and horizontal gene transfer have been described as being responsible for the host-specificity of the various formae speciales (ff. spp.) [19, 34, 35]. The predominant variation between *F. oxysporum* ff. spp. has been found in effector profiles on these conditionally dispensable, accessory chromosomes [16]. Previous studies have suggested that repeats and transposons on the accessory chromosomes increase the occurrence of point mutations, which expands diversity in virulence genes through higher mutation and duplication rates via chromosomal rearrangements and altered gene expression through chromatin restructuring [19, 35]. The identified LSX of *Fo koae* 44 in this study was transposon-rich and contained important wilt-inducing effector genes, *SIX1* and *SIX6*, providing evidence of one or more pathogen-specific, accessory chromosome(s). Interestingly, the identified LSX of *Fo* 170 was also transposon-rich, but it contained genes associated with root disease including the *PEP2* gene. The LSX identified here likely represent a combination of multiple lineage-specific chromosomes, but due to the methodologies used in this study, the LSX likely also contains assembly contigs that did not map to the reference, but could still belong on core chromosomes. However, a portion of DNA found within the LSX likely represents one or more lineage-specific chromosome(s) for both *Fo koae* 44 and *Fo* 170. Future karyotyping studies could determine presence and quantity of accessory chromosomes. Virulence-associated genes within the LSX are likely the determining factors that distinguishes pathogenicity of *Fo koae* 44 on koa.

Even though 1353 and 2369 predicted proteins were identified on the LSX of *Fo koae* 44 and *Fo* 170, respectively, the function of 58 and 50% of genes for *Fo koae* 44 and *Fo* 170, respectively, could not be determined from the available databases used for this study (Additional File 8). Perhaps these genes are highly specific to *Fo koae* 44 and *Fo* 170 and are possibly important for pathogenicity but have yet to be described. Since we did not conduct transcriptomic analyses in this study, we could not validate expression of these genes. Amongst the identified genes, most virulence-associated genes, secondary metabolites, and carbohydrate active enzymes were found to be similar for *Fo koae* 44 and *Fo* 170. Several well-characterized, virulence-associated genes (e.g., *Gpabc1*, *GzmetE*, *Tom1*, *Ace1*, *Mocdip1*, *Ftf2*, and *SGE1*) were identified in both *Fo koae* 44 and *Fo* 170. Interestingly, these genes suggest that *Fo koae* 44 and *Fo* 170 both have the capacity for host recognition and disease development, even though *Fo* 170 does not cause wilt disease on koa [36].

Of the 57 and 59 unique predicted genes from the LSX of *Fo koae* 44 and *Fo* 170, respectively, the major distinctions found in *Fo koae* 44 were a mitogen-activated protein kinase pathway (associated with genes *Fmk1*, *Ste11*, and *Ste7*), which were previously identified as important for host penetration and pathogenicity, and the *SIX1* and *SIX6* [37]. *SIX* genes have been well-characterized as important host-specific pathogenicity factors in various *F. oxysporum* formae speciales [15, 18]. *SIX1* and *SIX6* genes together have been reported in *F. oxysporum* f. sp. *lycopersici*, *F. oxysporum* f. sp. *melonis, F. oxysporum* f. sp. *niveum, F. oxysporum* f. sp. *canariensis*, *F. oxysporum* f. sp. *phaseoli* and *F. oxysporum* f. sp. *cubense* [3, 16, 20, 38–40]. With the banana pathogen, *F. oxysporum* f. sp. *cubense* (*Fo cubense*), tropical race 1 and tropical race 4 can be distinguished by *SIX* genes [41, 42]. The same *SIX1* and *SIX6* genes reported here in *Fo koae* 44 have been previously reported in *Fo cubense* race 1 [5]. *SIX* genes are surrounded by repeat- and transposon-rich regions that increase the chances of mutation and duplication of these important effectors [19]. Our results concur with these previous observations because we observed the localization of the two *SIX* genes amongst the transposon-rich LSX of *Fo koae* 44 [29]. These *SIX* genes, in conjunction with their associated transcription factor (*SGE1*) and the mitogen-activated protein kinase pathway, may be key characteristics that contribute to pathogenicity on koa.

Although *Fo* 170 lacked *SIX* genes, a stronger putative association was observed for secondary metabolite production and transport. The identification of the trehalose, putrescine, cyclodipeptide, and monooxygenase genes in *Fo* 170 signified that toxin production may be more important for disease development on a potential non-koa host of *Fo* 170. Interestingly, another secreted peptide, PEP2 (PHI:224), was identified as exclusive to *Fo* 170, and it has been found to be important for development of root rot on pea. These results also suggest that while *Fo* 170 does not cause koa wilt disease, it may be a pathogen on another host. To differentiate and monitor the koa wilt pathogen from its non-pathogenic counterparts, pathogen-specific primers were developed.

Pathogen-specific primers have previously been shown to be effective at detecting and monitoring pathogenic strains of fungi [43–47], and they are powerful tools in disease management, including disease resistance-breeding programs. Similar to recent studies, developed primers were based on unique non-coding sequences and exclusive predicted genes of *Fo koae* 44 [43–45]. To reduce the risk of false positive amplification of other *F*. *oxysporum* formae speciales isolated from koa, we chose to develop primers that would amplify genes unique to *Fo koae* 44 that did not have identified gene ontology terms.

Due to the importance of lineage-specific DNA and the virulence-associated genes found within, the LSX was targeted for primer development to detect the presence of the lineage-specific DNA that contains putative genes necessary for development of koa wilt disease [3, 16]. We observed that primer pair P6 developed for the LSX was more reliable to distinguish pathogenic isolates than unique genes identified on the “core” chromosomes, because no non-target amplification of DNA from characterized non-pathogenic isolates was observed with the LSX primer pair (P6). Of the 12 identified *rpb2* haplotypes, primer pair P4 resulted in target amplification of three haplotypes (Hap_1, Hap_3, and Hap_4), but primer pair P6 produced target amplification of only one haplotype (Hap_3)*.* These results indicate that isolates in haplotype Hap_3 may possess some putative lineage-specific DNA necessary for development of koa wilt disease. DNA of haplotypes that only amplified with primer pairs P4, may contain a homologous gene in their “core” chromosomes that may not be required for virulence that is also shared with other *Fusarium* spp.

The LSX primer pair (P6) resulted in amplification of DNA from only one *rpb2* haplotype which suggests that it may be too specific to amplify all highly virulent isolates within the *Fo koae* population; however, primer pair P6 produced amplified product for all pathogenic *Fo koae* isolates used in HARC’s disease resistance breeding program. The seven *Fo koae* isolates used in HARC’s disease resistance breeding program were collected on Hawai‘i (**44** and 166), Kauai (76, 77, 78, and 79), and Oahu (90); DNA from all of these isolates was amplified with both developed primer pairs [23, 48]. For the ongoing greenhouse pathogenicity trials, these highly virulent isolates (**44**, 77, 78, and 79) and three moderately virulent isolates (76, 90, and 166) are combined evenly by weight and used in combination as a soil inoculum [23]. This methodology (mixing of inoculum from multiple isolates), may allow anastamoses and/or asexual genetic recombination to occur between isolates. Hypothetically, the putative lineage-specific DNA used to develop our primers could have been transferred horizontally among isolates during the inoculation trials conducted by HARC [7], which could have contributed to the observed amplification of a single haplotype with the developed P6 primer pair. Targeted studies of the *Fo koae* population are needed to better understand the role of horizontal gene transfer in pathogenicity.

Future studies should include greenhouse virulence assays to characterize field-collected isolates that amplified with both the core primer pair (P4) and the LSX primer pair (P6). Population genomic analyses are needed to determine genetic relationships among pathogenic *Fo koae* and non-pathogenic *F. oxysporum* isolates across the Hawaiian archipelago. Such studies can determine if a direct comparison of one pathogen and one non-pathogen is sufficient for developing a primer to detect pathogenic *Fo koae*, or if whole genome sequences of multiple haplotypes are needed to develop more robust, pathogen-specific primers. In addition, karyotyping could validate the presence and quantity of the putative lineage-specific chromosome(s) of *Fo koae* 44.

## Conclusion

This study used whole genome analyses to predict virulence-associated genes that may be necessary for the development of koa wilt disease by *Fo koae*. This information was used to develop PCR primers for distinguishing highly virulent *Fo koae* isolates from non-pathogenic *F. oxysporum* isolates. Previously, the *SIX1* and *SIX6* genes have been characterized in other pathogens for their role in causing wilt diseases in other hosts, and it appears that these genes are also necessary for *Fo koae* to cause koa wilt disease. Genomic comparisons between *Fo koae* 44 and *Fo* 170 identified *Fo* 170-exclusive genes, including pathogenicity-related genes, which suggests that *Fo* 170 may be pathogenic to a different host without necessarily causing a wilt disease. Putative lineage-specific chromosomes were identified, but further research is needed to characterize and quantify these chromosomes. Pathogen-specific primers were developed that only amplified DNA of characterized, pathogenic isolates of *Fo koae*.

Because virulence assays have not yet been conducted on *Fo koae* haplotype isolates from the field collection, further research is needed to assess the robustness of the developed primers and determine if the predicted virulence-associated genes are required for pathogenicity. These results provide the framework for understanding the pathogen genes necessary for development of koa wilt disease and determining genetic variation of *Fo koae* populations across the Hawaiian Islands.

## Methods

### Greenhouse-characterized pathogenic and non-pathogenic isolates of *Fusarium oxysporum*

For this study, *Fusarium oxysporum* isolates that were previously screened for pathogenicity through greenhouse trials were provided by the Hawai‘i Agriculture Research Center (HARC) [48] (Table 2). These isolates were screened at HARC using methods described in Dudley et al. [48]. At 90 days post-inoculation of koa seedlings under greenhouse conditions, isolates were classified as non-pathogenic (< 10% mortality), low virulence (10–30% mortality), moderate virulence (30–70% mortality), or high virulence (70–100% mortality) (Table 2).

Twelve characterized pathogenic *F*. *oxysporum* isolates of high (**44**, 77, 78, and 79), moderate (8, 17, 20, 76, 90, 166, and 0540 K), and low (34) virulence, and five non-pathogenic (27, 53, 72, 81, and **170**) *F*. *oxysporum* isolates were used for reference in later analyses. Isolates 44, 76, 77, 78, 79, 90, and 166 are currently used in HARC’s disease resistance screening trials. Pathogenic isolate 44 and non-pathogenic isolate 170 were selected for whole genome sequencing.

### Whole genome sequencing and assembly

In preparation for DNA extraction, one isolate pathogenic to koa (*Fo koae* 44) and one non-pathogenic isolate (*Fo* 170) were first grown on ¼-strength PDA at 25 °C for 3 days. Hyphal tips were transferred to 100 ml of potato dextrose broth and shaken at 70 rpm for 7 days at room temperature. Mycelium was collected using a Buchner funnel and filtered through a Whatman® No. 1 filter paper under vacuum. Mycelium was placed in 2-ml tubes and frozen at 75 °C for 24 h prior to extraction. DNA was extracted using a cetyl trimethyl ammonium bromide (CTAB) extraction protocol adapted from Cubero et al. [49]. Tissue preparation was modified from the original protocol in that 0.1 g of frozen tissue was pulverized using a FastPrep-24^Tm^ (M.P. Biomedicals LLC, Santa Ana, CA, USA) at 5x speed for three 20-s runs. Between runs, samples were kept frozen using liquid nitrogen. On the third run, 750 μl of CTAB extraction buffer (1% w/v CTAB; 1 M NaC1; 100 mM Tris; 20 mM EDTA; 1% w/v polyvinyl polypyrolidone) was added to each sample. Samples were incubated for 30 min at 70 °C in a heat block. One volume of chloroform:isoamyl alcohol (24:1) was added to each sample and mixed on a shaker for 20 min then centrifuged at 10,000 x g for 5 min. Upper aqueous phase (600 μl) was transferred to a new 2-ml tube. Two volumes of Precipitation buffer (two volumes; 1200 μl) was added to sample, which was mixed in a shaker for 5 min, then centrifuged at 13,000 x g for 15 min. Supernatant was removed and re-suspended in 350 μl 1.5 M NaCl. To each sample, 2 μl of 10 mg/ml RNase was added, followed by incubation at 37 °C for 30 min. One volume of chloroform:isoamyl was added to each sample and mixed on a shaker for 20 min, then centrifuged at 10,000 x g for 5 min. Upper phase was transferred to a new 1.5-ml tube and 0.6 volume of ice-cold isopropanol was added to each sample. Samples were mixed by inversion and incubated at − 20 °C for 1 h. After incubation, samples were centrifuged at 14,000 x g for 5 min at room temperature. Supernatant was removed and 1 ml 70% ethanol was added to each sample. After 1-min incubation at room temperature, samples were centrifuged at 14,000 x g for 3 min at room temperature. Pellet was dried and resuspended in 50 μl TE buffer (10 mM Tris-HCl, 1 mM EDTA). Extracted DNA was gel electrophoresed in a 2% agarose gel and quantified using a Qubit™ fluorometer (Invitrogen, Carlsbad, CA, USA). A Zymoclean™ Gel DNA Recovery Kit (Zymo Research, Irvine CA, USA) and a Zymo clean and concentrate column were used to recover and purify the DNA. Extracted DNA was sent to Macrogen (Seoul, South Korea) for TruSeq Illumina shotgun sequencing with reads of 2x151bp.

De novo genome assemblies of both *Fo koae* 44 and *Fo* 170 were constructed using SPAdes 3.11.1 [50] using default parameters. The SPAdes genome assembly was assessed for quality using QUAST [51]. Contigs with than 100x coverage were removed, and the remaining contigs were checked for similarity with *Fusarium* spp. sequences using the National Center for Biotechnology Information (NCBI) BLAST to screen for presence of bacterial DNA in samples.

### Phylogenetic analysis

A whole-genome, maximum likelihood phylogeny was constructed to elucidate evolutionary relationships among *Fo koae* 44, *Fo* 170, other *Fusarium* spp., and other *F. oxysporum* formae speciales. REALPHY 112 [52] was used to generate whole genome alignments including the *F. oxysporum* f. sp. *lycopersici* reference genome and *Magnaporthe oryzae* (Both obtained from GenBank; Additional File 9). PhyML 3.1 [53] using bowtie2 2.3.4.2 [54] and the general time reversal (GTR) substitution model [55], was used to construct the whole genome phylogeny with bootstrap support (pseudoreplicates = 200) and *M. oryzae* as the outgroup. Whole genomes of *Fusarium* spp. and *F. oxysporum* formae speciales were obtained from the NCBI GenBank database (Additional File 9).

### Genome annotation and analysis

To determine overall sequence similarity of *Fo koae* 44 and *Fo* 170 isolates, average nucleotide identity was calculated using Pyani [56]. R package ‘coRdon’ was used to determine codon usage bias [57, 58]. TransposableELMT, a wrapper script for transposable element identification, and creation of a comprehensive repeat library using RepeatModeler, RepeatClassifier, LTR_finder, ltr_harvest, and TransposonPSI (http://transposonpsi.sourceforge.net) and subsequently RepeatMasker with this library were used to identify transposable elements and repeat-rich regions for both *Fo koae* 44 and *Fo* 170 [59–61].

To identify unique sequences of *Fo koae* 44 and *Fo* 170, putative chromosomes were constructed using a closely related reference genome, *F. oxysporum* f. sp. *lycopersici*. Paired reads of the genomes of *Fo koae* 44 and *Fo* 170 were trimmed using BBDuk (decontamination using kmers; part of BBTools package) [62]. The trimmed reads were mapped to the reference genome using Geneious (Geneious Prime 2019.2, http://www.geneious.com/) progressiveMauve genome mapper software at medium-low sensitivity [63, 64]. Low and high coverage sites were removed using Geneious’ built-in program. Variants and SNPs were identified using FreeBayes [65] within Geneious. Variant calling format (VCF) was exported for use in comparison of SNPs and indels between *Fo koae* 44 and *Fo* 170. Synteny maps and MUMmer alignments were made using the synteny mapping and analysis program (SyMAP) [66] to create putative chromosome contigs based on the reference genome.

The de novo assembled genomes were annotated for both *Fo koae* 44 and *Fo* 170 using the MAKER 2.31.8 annotation pipeline [67] with RepeatMasker 4.0.8 [60]. A *F. oxysporum*-specific repeat library was constructed using RepeatModeler 1.0.11 [68] to mask interspersed repeats and low complexity DNA sequences. Three gene predictors were used in the pipeline: GeneMark-ES [69], SNAP [70], and AUGUSTUS [71]. *Fusarium graminearum* was used as a species model for AUGUSTUS. To identify tRNA genes, tRNAscan-SE 1.3.1 was used with default settings.

Predicted transcripts (≥150 bp) and proteins (≥50 amino acids) were analyzed using five databases for analysis of putative genes: OrthoVenn2 for putative non-orthologous proteins [72], InterProScan for protein family domains [36], antiSMASH for putative secondary metabolites [73], dbCAN2 for putative carbohydrate-active enzymes [30], and PHI-base for putative virulence-associated proteins [74]. OrthoVenn2, antiSMASH, and dbCAN2 databases’ online servers were used to analyze the predicted genes at default settings. A local BLAST was used for InterProScan and the PHI-base database. Selection of putative proteins from PHI-base was based on a ≥ 85% grade (metric of combining the query coverage, e-value, and identity values for each hit with weights of 0.5, 0.25 and 0.25, respectively). Putative virulence-associated proteins were tested for exclusivity to *Fo koae* 44 by aligning these sequences to sequences obtained from the NCBI database using MUSCLE in Geneious [75] (Additional File 10).

### Primer development

Putative chromosomes from SyMAP were aligned using progressiveMauve [63] in Geneious. Using these alignments, unique sequences of *Fo koae* 44 were identified and selected. Also, predicted transcripts specific to *Fo koae* 44 were identified by comparing the predicted transcriptomes of *Fo koae* 44 and *Fo* 170 from the Maker annotation identified from Orthovenn2. *Fo koae* 44 predicted transcripts were further identified as unique by using a local BLAST against *Fo* 170 and the NCBI database. Sequences with no sequence similarity to sequences from *Fo* 170, other *Fusarium* spp., and *F. oxysporum* formae speciales were used to develop primer pairs. Primer3 [76] was used to develop PCR primers. *Fo koae* 44 unique sequences and predicted transcripts were used to generate ca. ten primer sets (forward and reverse primers) for each sequence and transcript.

Primers were tested for specificity using template DNA from four characterized highly virulent *Fo koae* isolates (isolates used in HARC greenhouse screening trials: **44**, 77, 78, and 79), seven characterized moderately virulent *Fo koae* isolates (HARC isolates 8, 17, 20, 76, 90, 166, and 0540 K), two characterized non-pathogenic *Fo* isolates (HARC isolates 27 and **170**), two *F. commune* isolates (HARC isolate 85 and Stewart collection FO21), and one *F. proliferatum* isolate (HARC isolate 1). Each 25-ul reaction contained 10 ng template DNA or sterile, molecular grade water for the negative control. Primers were tested using a Mastercycler ProS thermocycler (Eppendorf, Hamburg, Germany) at a program of 94 °C for 2 min, 30 cycles of 94 °C for 40 s, 59 °C for 40 s, and 72 °C for 30 s, and 72 °C for 5 min. PCR products for each primer set and template DNA were electrophoresed using a standard 1.5% agarose gel at 60 V for 60 min, and visualized using GelRed® (Biotium, Freemont, CA, USA).

### Field collection of *Fusarium* spp.

To assemble an expanded population of *F. oxysporum* isolates for *Fo koae* specific-primer tests, 14 collection sites were selected based on a previous survey conducted by HARC from 2004 to 2007 across four of the main Hawaiian Islands, including Kauai, Oahu, Maui, and Hawai‘i [77]. Sites for new isolate collections were selected based sites on where previously characterized pathogen isolates of moderate to high virulence were collected. In addition, selected collection sites were geographically distant from each other to increase the likelihood of collecting a more genetically diverse pathogen population. On Kauai, the three collection sites included Kokee, Kapa’a, and Hanalei (Princeville). On Oahu, three collection sites included Maunawili, Poamoho, and Kahana. On Hawai‘i, eight collection sites included Wood Valley, Pu‘u wa‘a wa‘a, Kona, Wung Ranch, Kaiwiki, Kalopa, Paauilo, and Volcano National Park. Root samples were collected from symptomatic trees at each site. Symptomatic segments of 4- to 5-mm thick roots exhibiting discoloration and black streaking, were targeted for isolation of *Fusarium* spp. Five fine roots and/or pencil-thick roots were plated on selective Komada media [78] for isolation of *Fusarium* spp. Plates were incubated at 25 °C for 3 days then checked for mycelial growth emanating from the root samples. Hyphal tips were taken from the radiating mycelium and transferred to ¼-strength PDA and incubated at 25 °C for another 3 days. Colony morphology was used to determine if the isolate was *Fusarium*. To further characterize these isolates to species, isolates were sequenced at the translation elongation factor 1 alpha (*tef1*) region using a Mastercycler ProS thermocycler and a PCR cycle program of 94 °C for 2 min, 40 cycles of 94 °C for 40 s, 58 °C for 40 s, and 72 °C for 30 s, and 72 °C for 5 min [79]. Products were run on a 1.5% agarose gel to visualize amplified PCR product using GelRed®.

### Genetic characterization of field-collected isolates

To determine the genetic relationships among the field-collected isolates and the previously characterized pathogenic and non-pathogenic isolates, *F. oxysporum* isolates, based of *tef1* data. Isolates were selected from separate, individual trees. The selected isolates were then sequenced at the RNA polymerase II second largest subunit (*rpb2*) [80] using previous methods above for *tef1*. *Rpb2* sequences of both characterized and field-collected isolates were aligned using MUSCLE alignment in Geneious [75]. A statistical parsimony (TCS) haplotype network was constructed based on this *rpb2* sequence data using PopART with a 95% genetic cut-off [81]*.* These isolates were tested with the developed *Fo koae*-specific primers to identify haplotypes that may contain the putative lineage-specific DNA identified from *Fo koae* 44.

## Supplementary information

**Additional file 1. **Whole genome maximum likelihood phylogeny based on *Fusarium* spp. and *F. oxysporum* (*Fo*) formae speciales. Pathogenic isolate *F. oxysporum* f. sp. *koae* (*Fo koae* 44) indicated in red and non-pathogenic isolate *F. oxysporum* (*Fo* 170) indicated in blue. Bootstrap = 100.

**Additional file 2. **Putative biological, molecular, and cellular function of the predicted non-orthologous proteins identified as unique to the pathogenic isolate of *Fusarium oxysporum* f. sp. *koae* (*Fo koae* 44) when compared to non-pathogenic isolate of *F. oxysporum* (*Fo 170*). Identified function is based on gene ontology (GO) terms.

**Additional file 3. **Putative biological process and molecular function of the predicted non-orthologous proteins identified as unique to the non-pathogenic *Fusarium oxysporum* isolate (*Fo* 170) when compared to the pathogenic isolate of *F. oxysporum* f. sp. *koae* (*Fo koae* 44). Function is based on gene ontology (GO) terms.

**Additional file 4. **Copy number of secondary metabolite genes including nonribosomal peptide synthetases (NRPS), type 1 and type 3 polyketide synthases (T1PKS and T3PKS), terpenes, and indoles for the pathogenic *Fusarium oxysporum* f. sp. *koae* isolate (*Fo koae* 44, red bars) and non-pathogenic *F*. *oxysporum* isolate (*Fo* 170, blue bars).

**Additional file 5. A)** Pathogen-specific PCR primers designed from unique genes and sequences in genome of *Fusarium oxysporum* f. sp. *koae* 44. Isolates were confirmed for pathogen specificity through PCR testing on greenhouse-characterized pathogenic and non-pathogenic isolates provided by the Hawai’i Agriculture Research Center. **B)** NCBI BLAST results of primer pairs P4 (designed to amplify “core” chromosome 2) and P6 (designed to amplify the putative lineage-specific DNA, LSX, of *Fo koae* 44).

**Additional file 6. **Results of 2018 field collection showing number of *Fusarium* isolates collected from symptomatic *Acacia koa*.

**Additional file 7. **Distribution and virulence ratings for 15 characterized and 85 uncharacterized *Fusarium oxysporum* isolates collected from *Acacia koa* roots used for haplotype and primer amplification analysis.

**Additional file 8. **Proportion of predicted genes with putative function of the lineage specific DNA (LSX) of the *Acacia koa* wilt pathogen *Fusarium oxysporum* f. sp. *koae* (*Fo koae* 44) and the *F. oxysporum* (*Fo* 170) isolate characterized as non-pathogenic to *A. koa*. Both isolates had similar proportions of transposons, repeats, carbohydrate active enzymes (CAZymes), and genes identitifed through gene ontology terms and protein family as having a biological, molecular, or cellular function. *Fo koae* 44 had a higher proportion of virulence associated genes and *Fo* 170 had a higher proportion of secondary metabolite genes on the LSX.

**Additional file 9. **Whole genome sequences of *Fusarium* spp. and formae speciales of *F. oxysporum* retrieved from NCBI with GenBank or RefSeq accession numbers. These genomes were used to make the whole genome phylogeny.

**Additional file 10. **Secreted in xylem (*SIX*) gene sequences of formae speciales of *Fusarium oxysporum* (*Fo*) retrieved from NCBI with GenBank accession numbers. Sequences used to compare identified *SIX* genes in pathogenic isolate *F. oxysporum* f. sp. *koae* 44.

**Additional file 11.** GenBank accession numbers and collection locations for all isolates used in this study.

## Data Availability

Cultures used in this study are stored at Colorado State University Stewart laboratory culture collection and are available upon request. All sequences have been deposited into National Center for Biotechnology Information (NCBI) BioPoject, BioSample, Nucleotide and GenBank and accession numbers are listed in Additional File 5B and Additional File 11. Site information including GPS locations, where available, are listed in Additional File 7 and Additional File 11 for each isolate. GenBank accession numbers of isolates used for comparison of secreted in xylem genes are listed in Additional File 10. GenBank accession numbers for reference genomes and genomes used for whole genome comparisons are listed in Additional File 9.

## Abbreviations

*Fo koae*: *Fusarium oxysporum* f. sp. *koae*
*Fo*: *Fusarium oxysporum*
koa: *Acacia koa*
*rpb2*: RNA polymerase II second largest subunit
*tef1*: Translation elongation factor 1 alpha
HARC: Hawai‘i Agriculture Research Center
LSX: Putative lineage-specific DNA
DNA: Deoxyribonucleic acid
Hap: Haplotype
*SIX*: Secreted in xylem
ANIm: Average nucleotide identity
GO: Gene ontology
NRPS: Non-ribosomal peptide synthetases
ABC: ATP-binding cassette
PCR: Polymerase chain reaction
CTAB: Cetyl trimethyl ammonium bromide
GTR: General time reversal
NCBI: National Center for Biotechnology Information
SNP: Single nucleotide polymorphism
VCF: Variant calling format
SyMAP: Synteny mapping and analysis program
