# Supplementary material for: Whole genome analysis of the koa wilt pathogen (Fusarium oxysporum f. sp. koae) and the development of molecular tools for early detection and monitoring
Source: BMC Genomics. 2020 Nov 4;21:764. doi: 10.1186/s12864-020-07156-y (PMC7640661; doi:10.1186/s12864-020-07156-y)
Supplement: Supplementary file 1 — Additional file 1. Whole genome maximum likelihood phylogeny based on Fusarium spp. and F. oxysporum (Fo) formae speciales. Pathogenic isolate F. oxysporum f. sp. koae (Fo koae 44) indicated in red and non-pathogenic isolate F. oxysporum (Fo 170) indicated in blue. Bootstrap = 100. [file 12864_2020_7156_MOESM1_ESM.pdf]

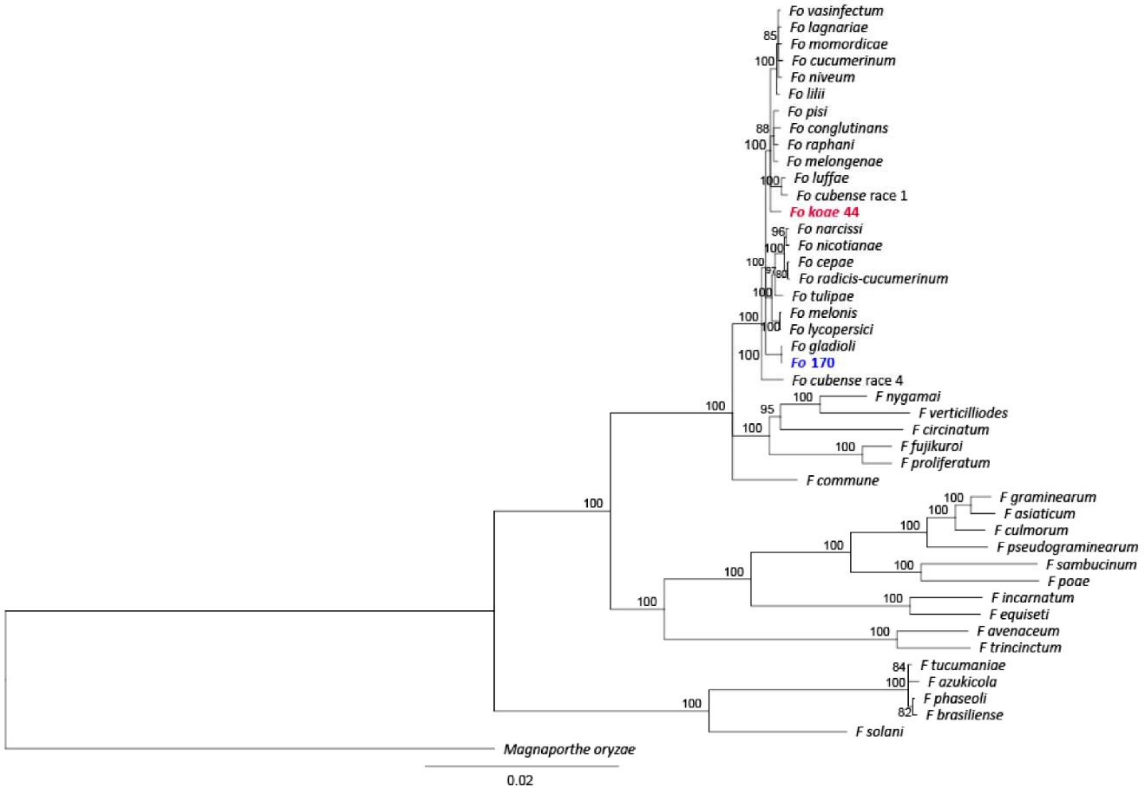

Additional File 1 – Whole genome maximum likelihood phylogeny based on *Fusarium* spp. and *F. oxysporum* (Fo) formae speciales. Pathogenic isolate *F. oxysporum* f. sp. *koae* (Fo koae 44) indicated in red and non-pathogenic isolate *F. oxysporum* (Fo 170) indicated in blue. Bootstrap = 100
