# Supplementary material for: Whole genome analysis of the koa wilt pathogen (Fusarium oxysporum f. sp. koae) and the development of molecular tools for early detection and monitoring
Source: BMC Genomics. 2020 Nov 4;21:764. doi: 10.1186/s12864-020-07156-y (PMC7640661; doi:10.1186/s12864-020-07156-y)
Supplement: Supplementary file 2 — Additional file 2. Putative biological, molecular, and cellular function of the predicted non-orthologous proteins identified as unique to the pathogenic isolate of Fusarium oxysporum f. sp. koae (Fo koae 44) when compared to non-pathogenic isolate of F. oxysporum (Fo 170). Identified function is based on gene ontology (GO) terms. [file 12864_2020_7156_MOESM2_ESM.pdf]

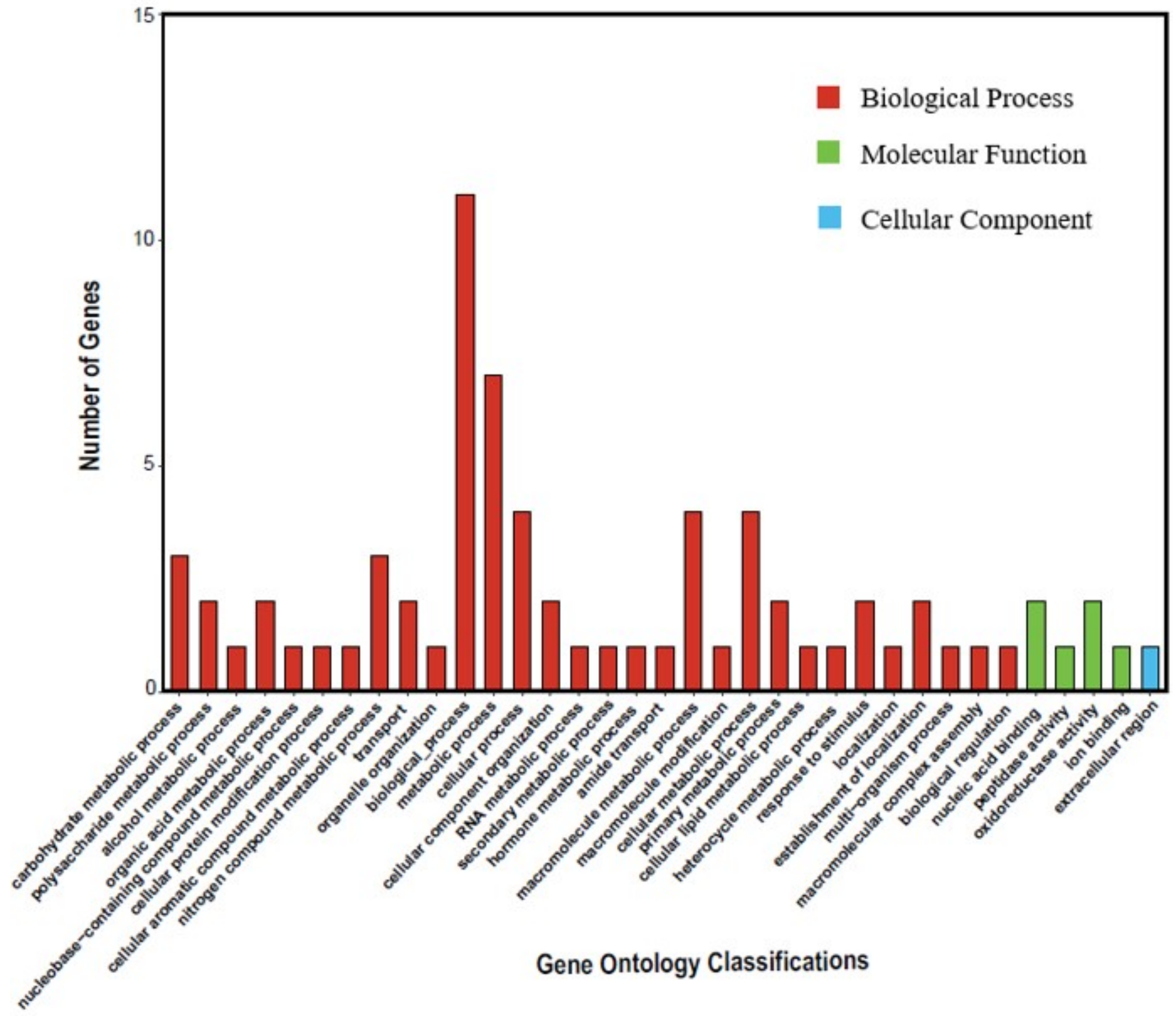

Additional File 2 – Putative biological, molecular, and cellular function of the predicted nonorthologous proteins identified as unique to the pathogenic isolate of *Fusarium oxysporum* f. sp. *koae* (*Fo koae* 44) when compared to non-pathogenic isolate of *F. oxysporum (Fo* 170). Identified function based on gene ontology (GO) terms.
