# Supplementary material for: Whole genome analysis of the koa wilt pathogen (Fusarium oxysporum f. sp. koae) and the development of molecular tools for early detection and monitoring
Source: BMC Genomics. 2020 Nov 4;21:764. doi: 10.1186/s12864-020-07156-y (PMC7640661; doi:10.1186/s12864-020-07156-y)
Supplement: Supplementary file 3 — Additional file 3. Putative biological process and molecular function of the predicted non-orthologous proteins identified as unique to the non-pathogenic Fusarium oxysporum isolate (Fo 170) when compared to the pathogenic isolate of F. oxysporum f. sp. koae (Fo koae 44). Function is based on gene ontology (GO) terms. [file 12864_2020_7156_MOESM3_ESM.pdf]

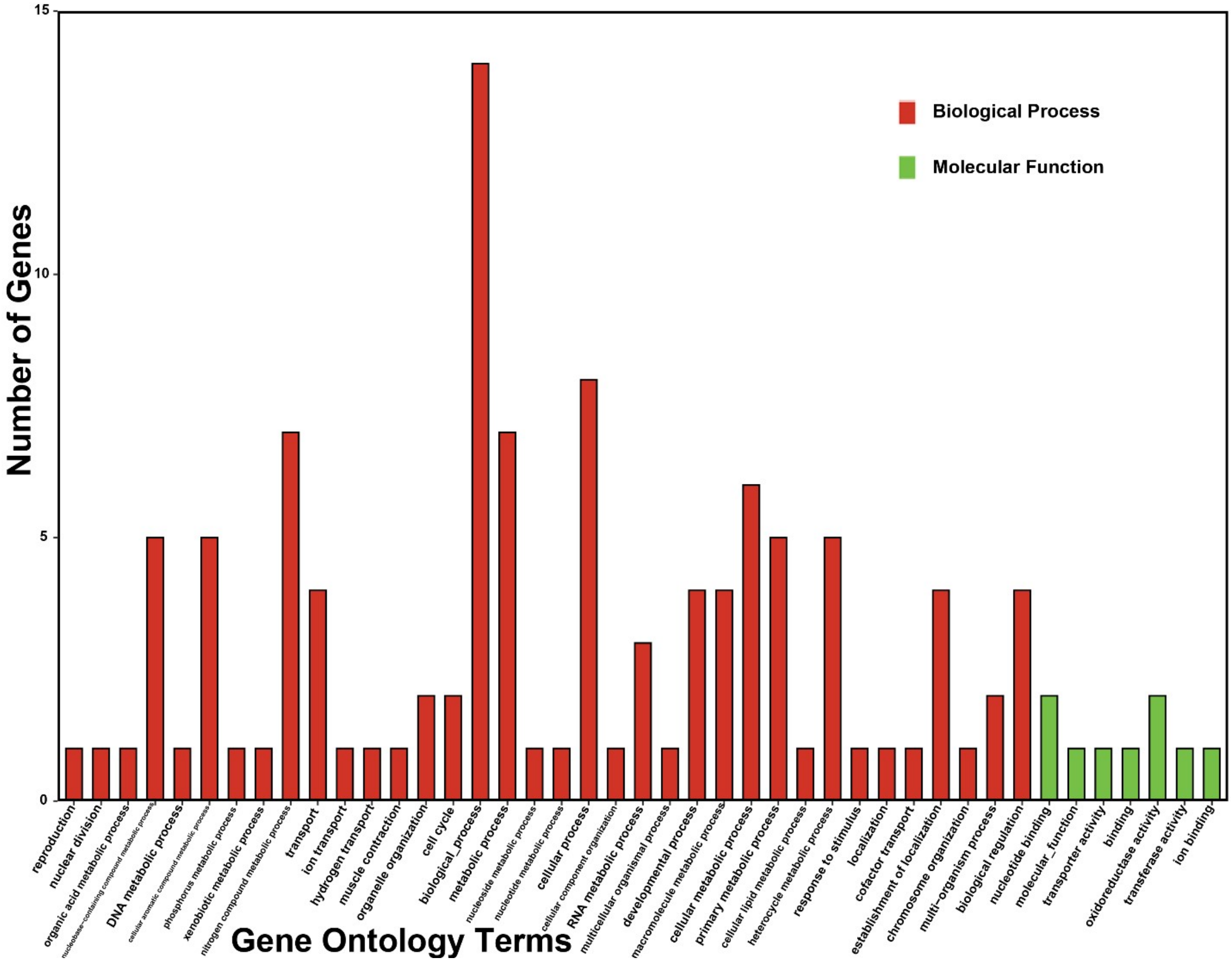

Additional File 3 – Putative biological process and molecular function of the predicted nonorthologous proteins identified as unique to the non-pathogenic *Fusarium oxysporum* isolate (*Fo* 170) when compared to the pathogenic isolate of *F. oxysporum* f. sp. *koae* (*Fo koae* 44). Function based on gene ontology (GO) terms.
