# Supplementary material for: Whole genome analysis of the koa wilt pathogen (Fusarium oxysporum f. sp. koae) and the development of molecular tools for early detection and monitoring
Source: BMC Genomics. 2020 Nov 4;21:764. doi: 10.1186/s12864-020-07156-y (PMC7640661; doi:10.1186/s12864-020-07156-y)
Supplement: Supplementary file 4 — Additional file 4. Copy number of secondary metabolite genes including nonribosomal peptide synthetases (NRPS), type 1 and type 3 polyketide synthases (T1PKS and T3PKS), terpenes, and indoles for the pathogenic Fusarium oxysporum f. sp. koae isolate (Fo koae 44, red bars) and non-pathogenic F. oxysporum isolate (Fo 170, blue bars). [file 12864_2020_7156_MOESM4_ESM.pdf]

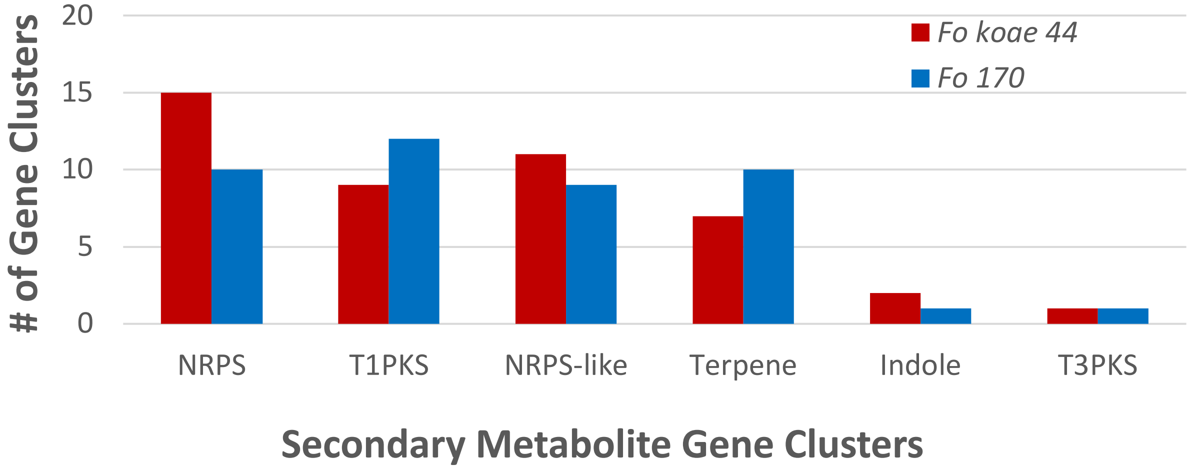

Additional File 4. Copy number of secondary metabolite genes including nonribosomal peptide synthetases (NRPS), type 1 and type 3 polyketide synthases (T1PKS and T3PKS), terpenes, and indoles for pathogenic *Fusarium oxysporum* f. sp. *koae* isolate (*Fo koae* 44, red bars) and non-pathogenic *F*. *oxysporum* isolate (*Fo* 170, blue bars).
