# Supplementary material for: Whole genome analysis of the koa wilt pathogen (Fusarium oxysporum f. sp. koae) and the development of molecular tools for early detection and monitoring
Source: BMC Genomics. 2020 Nov 4;21:764. doi: 10.1186/s12864-020-07156-y (PMC7640661; doi:10.1186/s12864-020-07156-y)
Supplement: Supplementary file 5 — Additional file 5. A) Pathogen-specific PCR primers designed from unique genes and sequences in genome of Fusarium oxysporum f. sp. koae 44. Isolates were confirmed for pathogen specificity through PCR testing on greenhouse-characterized pathogenic and non-pathogenic isolates provided by the Hawai’i Agriculture Research Center. B) NCBI BLAST results of primer pairs P4 (designed to amplify “core” chromosome 2) and P6 (designed to amplify the putative lineage-specific DNA, LSX, of Fo koae 44). [file 12864_2020_7156_MOESM5_ESM.pdf]

Additional File 5 A) Pathogen-specific PCR primers designed from unique genes and sequences in genome of *Fusarium oxysporum* f. sp. *koae* 44. Isolates were confirmed for pathogen specificity through PCR testing on greenhouse characterized pathogenic and nonpathogenic isolates provided by the Hawai'i Agriculture Research Center.

| **Primer Description** | **Primer Sequence** | **Chromosome** | **Putative Function** |
|---|---|---|---|
| P4 F<br>P4 R | TGTGGCCGCCTTGCAATAAC<br>GGTTTGTCCAACACACCCGT | 2 | Transcription Factor (25% identity)+ |
| P6 F<br>P6 R | GGCGCCAGCGTTTAATGGAA<br>ATTGGGCCTCCTTCGATCCG | LSX | Saponin detoxification (57% identity)+ |

*Pfam identified putative function

+PHI-base hit with low identity to genes that were identified as unaffecting pathogenicity

Additional File 5 B) NCBI BLAST top hits of non-target *Fusarium* spp. results of primer pairs P4 (designed to amplify "core" chromosome 2) and P6 (designed to amplify the LSX of *Fo koae* 44). Primer pair P4 had a 100% hit to the *F. xyrophilum* genome with a product size of 103bp at 3 loci suggesting the "core" designed primer pair is less specific than the LSX primer pair and needs to be tested on more diverse *Fusarium* spp.

| **Primer** | **Primer Size (nucleotides)** | **Primer Direction** | **Non-Target *Fusarium* spp.** | **Accession Number** | **Identities** |
|---|---|---|---|---|---|
| P4 | 20 | Forward | *F. xyrophilum* | VYXA01002211.1 | 20 |
| P4 | 20 | Reverse | *F. xyrophilum* | VYXA01002211.1 | 20 |
| P4 | 20 | Forward | *F. xyrophilum* | VYWZ01000697.1 | 20 |
| P4 | 20 | Reverse | *F. xyrophilum* | VYWZ01000697.1 | 20 |
| P4 | 20 | Forward | *F. xyrophilum* | VYWY01001448.1 | 20 |
| P4 | 20 | Reverse | *F. xyrophilum* | VYWY01001448.1 | 20 |
| P4 | 20 | Forward | *F. graminearum* | LT222054.1 | 15 |
| P4 | 20 | Reverse | *F. coffeatum* | XM_031161801.1 | 19 |
| P4 | 20 | Forward | *F. fujikuroi* | CP023098.1 | 15 |
| P4 | 20 | Reverse | *F. fujikuroi* | CP023091.1 | 14 |
| P4 | 20 | Forward | *F. culmorum* | LT598660.1 | 15 |
| P4 | 20 | Reverse | *F. culmorum* | LT598660.1 | 14 |
| P6 | 20 | Forward | *F. culmorum* | LT598660.1 | 19 |
| P6 | 20 | Reverse | *F. culmorum* | LT598660.1 | 14 |
| P6 | 20 | Forward | *F. fujikuroi* | CP023093.1 | 13 |
| P6 | 20 | Reverse | *F. fujikuroi* | CP023094.1 | 15 |
| P6 | 20 | Forward | *F. fujikuroi* | CP023105.1 | 13 |
| P6 | 20 | Reverse | *F. fujikuroi* | CP023106.1 | 15 |
| P6 | 20 | Forward | *F. coffeatum* | XM_031159536.1 | 14 |
| P6 | 20 | Reverse | *F. graminearum* | LT222056.1 | 17 |
| P6 | 20 | Forward | *F. venenatum* | LN649231.1 | 13 |
| P6 | 20 | Reverse | *F. venenatum* | LN649231.1 | 16 |
| P6 | 20 | Forward | *F. pseudograminearum* | XM_009257897.1 | 13 |
| P6 | 20 | Reverse | *F. pseudograminearum* | XM_009254468.1 | 15 |
