# Supplementary material for: Whole genome analysis of the koa wilt pathogen (Fusarium oxysporum f. sp. koae) and the development of molecular tools for early detection and monitoring
Source: BMC Genomics. 2020 Nov 4;21:764. doi: 10.1186/s12864-020-07156-y (PMC7640661; doi:10.1186/s12864-020-07156-y)
Supplement: Supplementary file 6 — Additional file 6. Results of 2018 field collection showing number of Fusarium isolates collected from symptomatic Acacia koa. [file 12864_2020_7156_MOESM6_ESM.pdf]

Additional File 6. Number of *Fusarium* isolates collected in 2018 from symptomatic *Acacia koa* at sites located on different Hawaiian Islands.

**Kauai**			
**Site**	**Number of Trees Sampled**	**Number of Trees Positive for *Fusarium***	**Number of *Fusarium* Isolates per Site**
Hanalei	11	6	8
Kokee	20	13	37
Kapa'a	1	0	0

**Oahu**			
**Site**	**Number of Trees Sampled**	**Number of Trees Positive for *Fusarium***	**Number of *Fusarium* Isolates per Site**
Kahana	11	6	9
Maunawili	12	12	64
Poamoho	8	8	27

**Hawai'i**			
**Site**	**Number of Trees Sampled**	**Number of Trees Positive for *Fusarium***	**Number of *Fusarium* Isolates per Site**
Wood Valley	20	16	57
Pa’auilo	14	11	48
Kalopa	10	10	60
Kaiwiki	9	4	4
Kona	1	1	1
Pu'uwa'awa'a	5	5	19
Volcano National Park	15	7	21
Wung Ranch	5	1	4
