# Supplementary material for: Whole genome analysis of the koa wilt pathogen (Fusarium oxysporum f. sp. koae) and the development of molecular tools for early detection and monitoring
Source: BMC Genomics. 2020 Nov 4;21:764. doi: 10.1186/s12864-020-07156-y (PMC7640661; doi:10.1186/s12864-020-07156-y)
Supplement: Supplementary file 8 — Additional file 8. Proportion of predicted genes with putative function of the lineage specific DNA (LSX) of the Acacia koa wilt pathogen Fusarium oxysporum f. sp. koae (Fo koae 44) and the F. oxysporum (Fo 170) isolate characterized as non-pathogenic to A. koa. Both isolates had similar proportions of transposons, repeats, carbohydrate active enzymes (CAZymes), and genes identitifed through gene ontology terms and protein family as having a biological, molecular, or cellular function. Fo koae 44 had a higher proportion of virulence associated genes and Fo 170 had a higher proportion of secondary metabolite genes on the LSX. [file 12864_2020_7156_MOESM8_ESM.pdf]

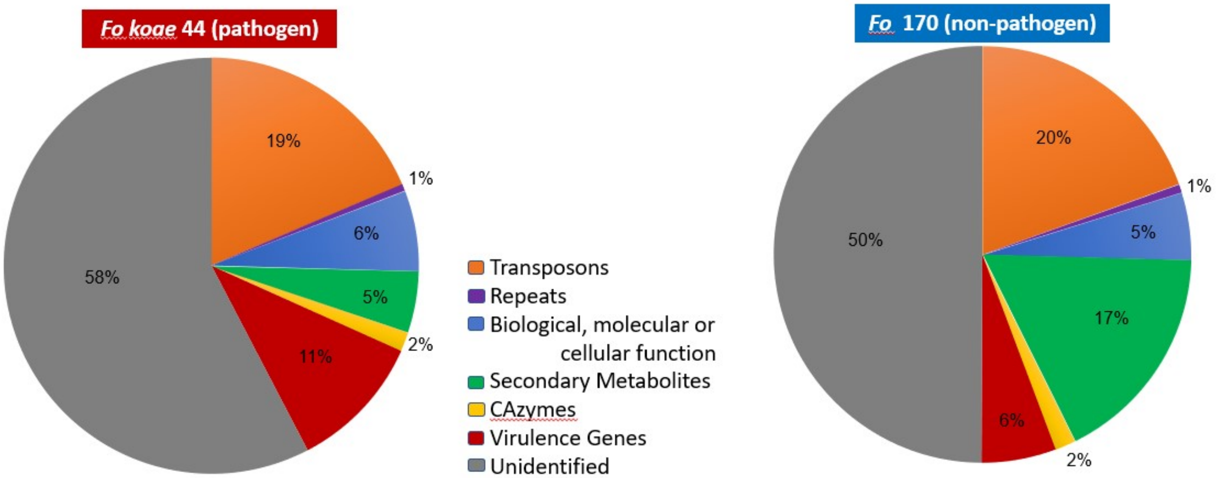

Additional File 8. Proportion of predicted genes with putative function of the lineage specific DNA (LSX) of the *Acacia koa* wilt pathogen *Fusarium oxysporum* f. sp. *koae* (*Fo koae* 44) and the *F. oxysporum* (*Fo* 170) isolate characterized as non-pathogenic to *A. koa*. Both isolates had similar proportions of transposons, repeats, carbohydrate active enzymes (CAZymes), and genes identified through gene ontology and protein families. *Fo koae* 44 had a higher proportion of virulence associated genes and *Fo* 170 had a higher proportion of secondary metabolite genes on the LSX.
