# Supplementary material for: Whole genome analysis of the koa wilt pathogen (Fusarium oxysporum f. sp. koae) and the development of molecular tools for early detection and monitoring
Source: BMC Genomics. 2020 Nov 4;21:764. doi: 10.1186/s12864-020-07156-y (PMC7640661; doi:10.1186/s12864-020-07156-y)
Supplement: Supplementary file 9 — Additional file 9. Whole genome sequences of Fusarium spp. and formae speciales of F. oxysporum retrieved from NCBI with GenBank or RefSeq accession numbers. These genomes were used to make the whole genome phylogeny. [file 12864_2020_7156_MOESM9_ESM.pdf]

Additional File 9. GenBank or RefSeq accession numbers for *Fusarium* spp. and formae speciales of *F. oxysporum* genomes used to make the whole genome phylogeny.

Species	Genbank Accession/ RefSeq
Fusarium asiaticum	LHTY00000000.1
F. avenaceum	JQGE00000000.1
F. azukicola	MAEG00000000.1
F. Brasiliense	MAEC00000000.1
F. circinatum	CM010400; AYJV00000000
F. commune	BCHB00000000.1
F. culmorum	FJUU00000000.1
F. equiseti	QOHM00000000.1
F. fujikuroi	GCF900079805.1
F. graminearum	AACM00000000.1
F. incarnatum	RBJE00000000.1
F. nygamai	LBNR00000000.1
F. phaseoli	MAEB00000000.1
F. pininemorale	NFZR00000000.1
F. poae	LYXU00000000.1
F. proliferatum	FJOF00000000.1
F. pseudograminearum	GCF_000303195.2
F. sambucinum	LSRD01000000.1
F. solani	NGZQ01000000.1
F. tricinctum	OVTS02000000.1
F. tucumaniae	MAED01000000.1
F. verticillioides	NC_031675.1
Fusarium oxysporum (*Fo*) *47*	AFMM01000000.1
Fo f. sp. *cepae*	CM010800.1
Fo f. sp. *conglutinans*	JH658799.1
Fo f. sp. *cubense* race 1	KB729921.1
Fo f. sp. *cubense* race 4	JH658272.1
Fo f. sp. *cucumerinum*	MABO00000000.1
Fo f. sp. *gladioli*	NJCK00000000.1
Fo f. sp. *lagnariae*	NJCJ00000000.1
Fo f. sp. *lilii*	NJCF00000000.1
Fo f. sp. *luffae*	NJCE00000000.1
Fo f. sp. *lycopersici*	AAXH00000000.1
Fo f. sp. *melongenae*	NJCC00000000.1
Fo f. sp. *melonis*	JH659329.1
Fo f. sp. *momordicae*	NJCB00000000.1
Fo f. sp. *narcissi*	NJCV00000000.1
Fo f. sp. *nicotianae*	NJBZ00000000.1

Fo f. sp. *niveum*	MALG00000000.1
Fo f. sp. *pisi*	JH650968.1
Fo f. sp. *radicis-cucumerinum*	CM008287.1
Fo f. sp. *raphanin*	JH658362.1
Fo f. sp. *tulipae*	NJBS00000000.1
Fo f. sp. *vasinfectum*	JH657918.1
Magnaporthe oryzae	NC_017844.1
