# Supplementary material for: Whole genome analysis of the koa wilt pathogen (Fusarium oxysporum f. sp. koae) and the development of molecular tools for early detection and monitoring
Source: BMC Genomics. 2020 Nov 4;21:764. doi: 10.1186/s12864-020-07156-y (PMC7640661; doi:10.1186/s12864-020-07156-y)
Supplement: Supplementary file 10 — Additional file 10. Secreted in xylem (SIX) gene sequences of formae speciales of Fusarium oxysporum (Fo) retrieved from NCBI with GenBank accession numbers. Sequences used to compare identified SIX genes in pathogenic isolate F. oxysporum f. sp. koae 44. [file 12864_2020_7156_MOESM10_ESM.pdf]

Additional File 10. GenBank accession numbers for *Fusarium oxysporum* formae speciales (*Fo*) retrieved from NCBI used to compare Secreted in Xylem (*SIX*) genes identified in pathogenic isolate *F. oxysporum* f. sp. *koae* 44.

SIX1			***SIX6***	
**Species**	**Genbank Accession**		**Species**	**Genbank Accession**
F. oxysporum (*Fo*) 37	KC296735.1		*Fo* f. sp. *cubense*	KX435008.1
Fo f. sp. *niveum*	KX435036.1		*Fo* f. sp. *vasinfectum*	KR855791.1
Fo f. sp. *fragariae*	KX435028.1		*Fo* f. sp. *radiciscucumerinum*	KR855755.1
Fo f. sp. *medicaginis*	KR855720.1		*Fo* f. sp. *niveum*	KR855756.1
Fo f. sp. *melonis*	KR811364.1		*Fo* f. sp. *melonis*	GQ268959.1
Fo f. sp. *conglutinans*	KR855720.1		*Fo* f. sp. *pisi*	KR855779.1
Fo f. sp. *lini*	KM893920.1		*Fo* f. sp. *cucumerinum*	KR855770.1
Fo f. sp. *canariensis*	MH616619.1		*Fo* f. sp. *passiflorae*	KR855782.1
Fo f. sp. *cubense*	KM893911.1		*Fo* f. sp. *lycopersici*	KR855786.1
Fo f. sp. *lycopersici*	KR855716.1		*Fo* f. sp. *phaseoli*	KP964967.1
